# Association of Physical Activity Level, Income and Environmental Factors Among Chinese Adults

**DOI:** 10.3389/fpubh.2022.946756

**Published:** 2022-06-30

**Authors:** Jingjuan Dong, Wei Liu

**Affiliations:** ^1^Physical Education College, Quanzhou Normal University, Quanzhou, China; ^2^Key Research Base of Humanities and Social Sciences in Colleges and Universities in Anhui Province–Quality Education Research Center for College Students of Anhui Xinhua University, Hefei, China; ^3^Department of Physical Education, Anhui Vocational and Technical College of Sports, Hefei, China

**Keywords:** physical activity, residents' income, environmental factors, intermediary effect, promotion

## Abstract

The purpose of this study was to evaluate associations between physical activity level and income and environmental factors among chinese adults. Through the empirical study of the survey data, it is found that: (1) after controlling for the relevant variables, residents' income has a positive impact on residents' participation in physical activities, and the regression coefficient is 0.541 (*P* < 0.01) (2) In the regression model after adding environmental variables, the regression coefficient of environmental variables increased from 0.401 (*P* < 0.01) to the highest 1.32 (*P* < 0.01) (3) Through the comparison of the KHB decomposition method, the intermediary effect of residents' income on environmental factors and participation in physical activities is 0.134 (*P* < 0.01), and the intermediary effect ratio is 27.01%. Suggestions: first, the government and society should actively provide public goods needed for physical activities to meet the needs of residents for physical activities; second, the government should strengthen the supply of sports facilities in residential areas, speed up the transformation of villages in cities and improve the living conditions in residential areas; third, through legislation to protect residents' income, improving income is not only to give citizens enough sense of security and happiness but also a way to improve residents' participation in physical activities.

## Introduction

For a long time, it has been mentioned in Chinese society that people participate in physical activities. Most people will intuitively think that this is a proprietary research in the field of sports and medicine, and they seldom think or explore from the perspective of behavioral science and social science. However, a large number of literatures at all times and in all over the world show that the research on physical activity participation behavior is not limited to this, but is closely related to sociology, economics and management. As the universal and eternal pursuit of all humankind, health is the starting point of all wellbeing. Promoting people's health has become the common governance goal of governments all over the world. In Chinese society, national health is one of the strategic paths to achieve “Healthy China 2030.” Physical activity, as an important means to increase national health and improve the quality of the whole population, is the main measure for the construction of “healthy China.” In the world, lack of physical activity has become the fourth fatal factor (1). At the same time, physical activity also plays an important role in people's health and socioeconomic development around the world. Some studies show that in 2013, due to a lack of physical activity, the global health care system spent US $53.8 billion, the public sector spent US $31.2 billion, the private sector spent US $12.9 billion and households lost US $9.7 billion and lost $13.7 billion in productivity (2). Physical activity has become a global issue and an important reference to measure people's livelihood.

In current Chinese society, there are many factors affecting residents' participation in physical activities. For example, site factors, leisure time factors and sports awareness factors. However, the existing studies basically support the correlation between site factors, leisure practical factors and sports awareness factors and physical activities. In fact, there is a relative lack of research on the impact of Chinese social residents' income and environmental factors on residents' physical activity participation. This paper attempts to study the impact of residents' income and environmental factors on their participation in physical activities. Many studies have verified the causal relationship between residents' income and physical activity from the perspective of socioeconomic status.

Many studies have verified the causal relationship between residents' income and physical activity from the perspective of socioeconomic status. Chen believes that there are significant differences in the socioeconomic status of Chinese residents participating in physical activities among different groups through research. He found that the possibility of participating in physical activities is reduced due to more housework in the group with lower socioeconomic status, while participation in physical activities is promoted due to long-term sedentary behavior in the group with higher socioeconomic status. In terms of specific individual structure influencing factors, individual differences in education years, income and occupation lead to differences in physical activities among people (3). The same empirical research conclusion has been verified again in other studies, that is, the analysis of occupation, education years and income and the frequency, time and intensity of physical activity participation. Through regression analysis, it was found that occupation, years of education and income have different effects on physical activity (4). Some studies have also found that there are correlations and differences between citizens' physical activity participation and residents' socioeconomic status in China. Among them, the increase in years of education will make residents' physical activity participation better than in groups with the same socioeconomic status, but there is no significant difference between groups of different occupational types (5). From the above representative research results of Chinese residents' participation in physical activities, the specific path of socioeconomic status affecting residents' participation in physical activities through individual income factors has temporal and spatial heterogeneity, which has important practical significance for the sustainable study of the impact of socioeconomic status on physical activities.

At present, China's urbanization process is developing rapidly. On the one hand, the development of urbanization has improved the lives of residents; on the other hand, the rapid development of urbanization has brought about a fast-paced life, which has also changed residents' physical activity participation, especially the increase in residents' motorized travel mode. The lack of necessary physical activities has made residents' physical activity time and probability much lower than before. This is similar to Zhang Wang's previous research view: through a large number of empirical studies, it is found that residents' individual physical activity participation is affected not only by individual physiological and psychological characteristics but also by external environmental pressure. Among them, the built environment of the city is found to be the key factor affecting urban residents' physical activity participation (6). There are also many experiences and achievements in the research of the urban built environment on physical activities. For example, the impact of the early built environment on physical activity participation is basically based on social ecological theory, mainly to examine the impact of the micro environment on physical activity participation (7). With the improvement and development of built environment measurement methods, a large number of empirical studies have found that there is also a correlation or causal relationship between urban form and physical activity (8). For example, reducing the residential density of residential areas, the surrounding environment of the community, the necessary infrastructure for life and traffic security will positively affect the physical activity level of residents (9). At the same time, foreign studies are just the opposite. In the survey objects, whether the basic demographic characteristics in the sample are controlled or not, the high residential density will indirectly improve the necessary walking activities in the residents' transportation journey (10), and the better the surrounding environment is, the more it is not conducive for residents to participate in physical activities. The reason is that the more beautiful the environment is, the more inconvenient the mixed land use is, and the connecting line of the road will become worse (11). It is not difficult to see that both at home and abroad have found that environmental pressure will have a certain impact on residents' physical activities. In fact, the premise of the environmental pressure around residents and the impact on residents' participation in physical activities is the result of the joint shaping of residents' psychology, behavior, society and economy. Its essence is the impact of residents' socioeconomic status on residents' individuals through the level of environmental pressure. It is also the complexity of this impact that makes researchers uncertain about the impact of the physical environment on residents' physical activity participation. This may be similar to the self-assessment type basically adopted in such investigations and studies, which may be biased due to the great influence of subjective pressure.

## Data and Variables

The research data of this paper come from the network survey data of the research group in 2021. The data survey is oriented to East China, including six provinces and one city, including Shanghai, Jiangsu, Zhejiang, Anhui, Fujian, Jiangxi and Shandong. The research group collected data on related physical activities, communities and families through the network questionnaire to summarize the development of social physical activities. It is of practical significance to explore relevant research with social scientific significance, promote the development of physical activity research, and provide data for regional development research. The survey data are based on the research supported by the fund project. There were 2,125 valid samples in 2021, including 28 variables. According to the needs of the research object, this study involves multiple variables, such as gender, age, living environment, physical activity, and education. In this study, after deleting missing values and outliers according to relevant needs in data processing, a total of 1998 valid data entered the analysis samples coefficient.

### Dependent Variable

The theme of this paper is the impact of residents' income and environmental factors on their participation in physical activity. Therefore, physical activity is the dependent variable of this study. In the relevant data of this study, there are relevant topics related to this variable. The specific question is as follows: have you participated in physical activities in your spare time in the past 6 months? The options are daily, several times a week, several times a month, several times a year, and never; 998 = do not know; 999 = refuse to answer. To ensure the validity of the research conclusion and the convenience of the data, we convert this variable into a continuous variable: 5 = daily; 4 = several times a week; 3 = several times a month; 2 = several times a year; 1 = never. A total of 998 and 999 samples were excluded from the study.

### Independent Variable

The database of this research project is a special social survey database about physical activity. For the independent variable of resident income, use the question “What was your total income in the previous year?” “To reduce the correlation error, we deal with income logarithmically”. In terms of environmental factors, the question “Does your community have enough physical activity venues and facilities?” The number of sites and facilities is set as the number of sites and facilities, and the number of facilities is set as the number of general facilities and the number of facilities. The number of facilities is set as the number of facilities to meet the basic needs. The number of facilities is set as the number of sites and facilities, and the number of facilities is set as the number of facilities to meet the basic needs.

### Other Variables

Other relevant variables in this study are gender. In this study, gender is transformed into category variables, in which female is 0 and male is 1. Educational variables: This study transforms educational variables into category variables, including 1 for junior middle school and below, 2 for senior high school and below, and 3 for University and above. Age variable. Age was set as a continuous variable in the study. The actual filling range of age in the questionnaire was 18–87 years old. Urban and rural variables, because there is an urban-rural dual structure in China, and the urban-rural dual structure affects residents' physical activity participation, are introduced in this study, and urban-rural variables are set as classified variables, in which rural is 1 and urban is 2. In fact, whether residents have jobs or not affects residents' income, lifestyle and interest. The descriptive statistics of specific variables are shown in Table 1.

**Table 1:** Descriptive statistics.

**Variable**	**Obs**	**Mean**	**Std. Dev**.	**Min**	**Max**
Physical activity	1998	1.791	1.461	1	5
Education	1998	1.449	0.831	1	3
Income	1998	8.641	4.231	0	16.118
Environment	1998	3.131	1.016	1	5
Gender	1998	0.481	0.487	0	1
Age	1998	35.063	14.231	18	87
Urban	1998	1.291	0.51	1	2

To analyse the effects of residents' income and environmental factors on residents' participation in physical activities. In this study, we used regression analysis and intermediary analysis to explore the relevant variables. At the same time, KHB analysis was used to test the intermediary role of residents' income in environmental factors and physical activities. Similar research methods include (12, 13).

## Result

In this study, the ordinal logit model was used for regression analysis. To better distinguish the impact of residents' income and environment on physical activities. In this study, four ordinal logit models were constructed for regression. As shown in Table 2, model 1 is the basic model, including three control variables: gender, urban and rural areas and age. Through model 1, it can be seen that gender, age and urban-rural differences have a significant impact on residents' physical activities. In fact, in most parts of China, residents' education affects residents' income and living environment. Therefore, we add the classified variable of education in model 2. Through model 2, we can see that there is a significant correlation between residents' education and physical activity, and the correlation coefficient increases from 0.891 (*P* < 0.01) to 1.378 (*P* < 0.01) Model 3 is a variable model for increasing residents' income, in which the variable coefficient of residents' income is 0.541 (*P* < 0.01) after controlling for relevant variables. Model 4 is the regression model after adding environmental variables, and the regression coefficient increases from 0.401 (*P* < 0.01) to the highest 1.32 (*P* < 0.01). In Table 3, this paper uses the KHB decomposition method proposed by Karlson to test the mediating factors of residents' income in environmental factors and outcome variables of participation in physical activities. The KHB decomposition method is used for the coefficient comparison between different models in the same sample. After comparison by the KHB decomposition method, the intermediary effect of residents' income is 0.134 (*P* < 0.01), and the intermediary effect ratio is 27.01%.

**Table 2:** Regression analysis model of residents' income, environmental factors and physical activities.

	**Model 1**	**Model 2**	**Model 3**	**Model 4**
	**PA**	**PA**	**PA**	**PA**
Gender	0.121^***^	0.481^***^	0.181^***^	0.169^**^
	(0.029)	(0.021)	(0.022)	(0.024)
Age	−0.012^***^	0.016^***^	0.041^***^	0.051^***^
	(0.002)	(0.002)	(0.001)	(0.001)
Urban	1.346^***^	0.981^***^	0.875^***^	0.789^***^
	(0.041)	(0.039)	(0.038)	(0.037)
**1bn.Edu**
2.Edu		0.891^***^	0.141^***^	0.081^***^
		(0.046)	(0.045)	(0.045)
3.Edu		1.378^***^	0.712^***^	0.198^***^
		(0.056)	(0.055)	(0.055)
Income			0.541^***^	0.231^***^
			(0.011)	(0.011)
**1bn.Environment**
2.Environment				0.401^***^
				(0.12)
3.Environment				0.771^***^
				(0.111)
4.Environment				0.781^***^
				(0.096)
5.Environment				1.032^***^
				(0.121)
Observations	1998	1998	1998	1998
Pseudo *R*^2^	0.089	0.101	0.152	0.189

*Standard errors are in parentheses*.

*** *p < 0.01,*

** *p < 0.05,*

*p < 0.1.

**Table 3:** Analysis of the intermediary effect of residents' income on environmental factors and physical activities.

**Intermediary variable**	**Independent variable**	**Total effect**	**Direct effect**	**Intermediary effect**	**Proportion of intermediary effect**
Income	Environment	0.496^***^	0.362^***^	0.134^***^	27.01%

*** *p < 0.01,*

**p < 0.05, *p < 0.1.

## Discussion

Through the results, we found that gender, age, urban and rural areas, education, income and environment have a significant impact on residents' physical activities. Among them, the influence of gender factors increases after adding the educational variable, which shows that education has a positive significance for men to participate in physical activities, or the concept of son preference still exists in Chinese society, and men are more likely to receive education than women, which affects women's participation in physical activities. Although educational factors play a positive role in residents' physical activity participation, this is similar to previous research conclusions; that is, individuals with higher educational levels are less likely to smoke, and smokers are more likely to quit smoking and exercise (14). The reason for this may be that educational achievement will have a great impact on many aspects of individual life in modern society, so it represents an important self-induced social identity (15). In fact, most studies on the impact of education on physical activity are carried out in Western society. Comparatively speaking, the attention of developing countries represented by China in this field is still very low. In Chinese society, the transaction of education often determines the level of income. It can be said that educational achievement is another embodiment of residents' income. Nevertheless, it can also be found that with the addition of residents' income and environmental variables, the difference between educational factors is narrowing, which shows that the impact of residents' social capital factors on residents' participation in physical activities in China will dilute educational factors. At the same time, through the change in the regression coefficient, we can also see that there are groups with more years of education and higher income in Chinese society, which are more likely to have a healthy life (16); that is, they are more likely to choose to participate in physical activities, which also shows the consistency between educational achievements and income increase in Chinese society. The same situation also occurs in the environment. The surrounding environment of residents will improve with the increase in income, and this environmental condition also encourages residents to participate in physical activities. It is not difficult to see that the income and environmental factors of residents in Chinese society can actively promote physical activity participation. However, the conclusion of the intermediary analysis shows that residents' income plays a partial intermediary role in environmental factors and participation in physical activities. In other words, the impact of environmental factors on residents' participation in physical activities is partly realized through the increase in residents' income, which also reflects that even if the surrounding environment is very superior, some people will not actively participate in physical activities, and the increase in personal income may promote their participation in physical activities, which is related to the fact that most of the participants in physical activities in China have money and leisure time. This also shows the complexity of the impact of residents' income and environment on participation in physical activities.

## Conclusion

Through the above four regression models and intermediary analysis, this paper discusses the impact of residents' income and environment on physical activities. Through the regression model, we can see that residents' income and environment have a positive correlation trend on physical activity participation, and residents' income plays an intermediary role in environmental factors and physical activity participation. That is, (1) with the increase in residents' income, the possibility of participating in physical activities will increase. (2) The higher the residents' satisfaction with the environment, the greater the possibility of participating in physical activities. (3) The impact of environmental factors on residents' participation in physical activities can be realized by increasing residents' income.

## Proposal

Therefore, this paper suggests the following: first, with the increase in Chinese residents' income, residents' demand for physical activities will show an increasing trend. It is suggested that the government and society actively provide the public goods needed for physical activities to meet residents' demand for physical activities. Second, the environmental factors around Chinese residential areas will promote their participation in physical activities. It is suggested that the government strengthen the supply of sports venues and facilities in residential areas, speed up the transformation of villages in cities and improve the living conditions in residential areas. Third, through legislation to protect residents' income, improving income is not only to give citizens enough sense of security and happiness but also a way to improve residents' participation in physical activities.

## Data Availability Statement

The raw data supporting the conclusions of this article will be made available by the authors, without undue reservation.

## Ethics Statement

The studies involving human participants were reviewed and approved by Anhui Vocational and Technical College of Sports. The patients/participants provided their written informed consent to participate in this study.

## Author Contributions

JD drafted the work and revised it critically for important intellectual content. WL contributed to the initial drafting of the manuscript. Both authors contributed to the article and approved the submitted version.

## Conflict of Interest

The authors declare that the research was conducted in the absence of any commercial or financial relationships that could be construed as a potential conflict of interest. The handling editor AI is currently organizing a Research Topic with the author WL.

## Publisher's Note

All claims expressed in this article are solely those of the authors and do not necessarily represent those of their affiliated organizations, or those of the publisher, the editors and the reviewers. Any product that may be evaluated in this article, or claim that may be made by its manufacturer, is not guaranteed or endorsed by the publisher.
